# Micro Defects on Diamond Tool Cutting Edge Affecting the Ductile-Mode Machining of KDP Crystal

**DOI:** 10.3390/mi11121102

**Published:** 2020-12-14

**Authors:** Shuo Zhang, Wenjun Zong

**Affiliations:** Center for Precision Engineering, Harbin Institute of Technology, Harbin 150001, China; shuozhang@hit.edu.cn

**Keywords:** tool-edge micro defects, SPH method, passivation method, chamfered edge, KDP crystal

## Abstract

As a soft-brittle material, the machined surface quality of potassium dihydrogen phosphate (KDP) crystal is heavily affected by the edge quality of the diamond cutting tool. However, nanoscale micro defects inevitably occur on the freshly sharpened tool edge, and the machining mechanism for KDP crystal remains unclear. Therefore, in this work, three types of tool-edge micro defects are classified according to their cross-sections, including the blunt-edge, crescent-edge, and flat-edge micro defects. Moreover, the smoothed particle hydrodynamics (SPH) method is employed to reveal the material removal mechanism of KDP crystal with consideration of different tool-edge micro defects, and the flat-edge micro defects are subdivided into flat edge A (similar to flank wear) and flat edge B (similar to chamfered edge) on the basis of their effects in machining. The simulation results indicate that the surfaces machined by crescent edge and flat edge A are unsmooth with large-size defects due to the disappearance of hydrostatic pressure beneath the cutting edge. As for the blunt edge and flat edge B, the machined surfaces are smooth with a favorable increment of hydrostatic pressure for processing brittle materials, which indicates that a solution to eliminate the tool-edge micro defects is necessary, e.g., the passivation method. For keeping the cutting edge as sharp as possible in removing the tool-edge micro defects completely by passivation, the effect of tool shank depression angles on the geometries of the passivated cutting edge is investigated, and a high-quality cutting edge with a micro chamfered edge is obtained after passivation at a depression angle of 60° and re-sharpening of the rake face. Finally, the tool cutting performance after passivation is validated through fly-cutting experiments of KDP crystal. The chamfered edge can produce the best defect-free surface with the minimum surface roughness.

## 1. Introduction

In diamond turning, except for the high-precision machine tool [1,2], super-stable machining environment [3,4], and material properties [5], the high-quality cutting tool is also an important factor that influences the machined surface quality [6,7,8]. As the most ideal material for the cutting tool used in ultra-precision machining [9,10], natural diamond has unmatched characteristics, such as super hardness, good resistance to abrasion, high strength, as well as low friction coefficient [11,12]. Moreover, the uniform crystal structure of natural diamond without internal grain boundaries makes the tool cutting edge reach the nanometer scale flatness and sharpness [13].

For achieving a high-quality cutting edge, various diamond tool-sharpening methods were developed, e.g., mechanical polishing [14], thermochemical polishing [15,16], ion beam polishing [17,18], chemically assisted mechanical polishing [19], and so forth. However, the extremely low removal rate of the chemical method and ion beam polishing is the key factor that restricts the application to the tool manufacturing industry. In fact, the traditional method, i.e., mechanical polishing, is always the most popular processing method for diamond cutting tools. In order to reveal the material removal mechanism in mechanical polishing of diamond cutting tools, Zong et al. [20] proposed a brittle–ductile transition mechanism considering the dynamic critical depths of cut in different directions on different planes. Moreover, a theory of dynamic critical tensile stress was further advanced, which can be regarded as a reference to estimate the effect of tool face orientation on the sharpened cutting edge radius [21]. An excellent diamond cutting tool oriented along (110)–(100) was finally polished, the cutting edge radius of which was sharpened down to 2–9 nm [22].

However, besides the tool sharpness (i.e., the cutting edge radius), the consistency of cutting edge quality is also a particularly important indicator. For example, micro breakage takes place inevitably in the brittle polishing mode, which will generate micro defects on the cutting edge and destroy the sharpness [23]. Even in the ductile polishing mode, micro defects may still occur on the cutting edge due to the minor change of the contact state between the rapid rotating scaife and the polished tool surface, which is enormously affected by the dynamic balance of the spindle, the vibration of the polishing setup [24], the surface quality of the scaife, the compression force in polishing, and so forth. Unfortunately, few studies have focused on the tool-edge micro defects and the possible effect on the machined surface quality. The reason is that measuring the extremely small cutting edge for a diamond tool is always a major challenge. As a conventional method, scanning electron microscopy (SEM) is a qualitative approach to evaluate the cutting edge [25]. However, when the cutting edge radius is less than 100 nm, the imaged edge becomes blurry. In order to accurately measure the cutting edge sharpness, Asai et al. [26] proposed a new kind of SEM measurement method by using two secondary electron detectors. The cutting edge shape can be obtained according to the difference of image signals from different SEM detectors.

In recent years, the high-resolution atomic force microscopy (AFM) technology had been developed. Generally, the AFM imaging of machined surfaces is an excellent way to obtain surface roughness and topography [27,28]. The AFM has also been applied to scan the nanoscale three-dimensional (3D) topography of the tool cutting edge directly [29,30]. Lucca et al. [31] firstly reported the measured edge profiles of new and worn diamond tools by AFM, taking the compensation of the cantilever tip radius into account. After indenting the tool cutting edge into a super-smooth surface of soft substrate [32], AFM was employed to measure the copy of the indentation profile to indirectly get the 3D topography of the cutting edge at the nano-precision level. Moreover, for a precision and fast measurement of the diamond tool cutting edge profiles, Gao et al. [33,34] designed an in situ hybrid measuring system composed of AFM and an optical alignment sensor. A focused laser beam is generated as a reference to make the AFM cantilever tip in alignment with the tool cutting edge precisely and quickly.

Moreover, to investigate the effect of the cutting edge radius, a lot of effort has been made. For instance, Hosseini et al. [35] used the molecular dynamics (MD) method to simulate the effect of the tool cutting edge radius on the contact state in nanomachining of single-crystal copper. Compared with a sharp tool, they found that a large fraction of atoms were compressed to pass through beneath the tool edge when using a blunt tool. The simulation results also indicated that with the increment of cutting edge radius, the principal cutting force *F_c_* is slightly increased, whereas the thrust cutting force *F_t_* is remarkably raised, which induces a higher compressive hydrostatic stress in a wider tool–chip contact length. As for the effect of the cutting edge radius on machining brittle materials, Arefin et al. [36,37] reported that if the cutting edge radius does not exceed a certain upper limit, and the undeformed chip thickness in processing is smaller than the cutting edge radius, the machined surface without cracks can be achieved. Through cutting experiments with appropriate processing parameters, they found that the upper limit of the cutting edge radius for the ductile mode machining of silicon wafer is around 807 nm. Moreover, Fang et al. [38] reported that when the undeformed chip thickness is comparable with the cutting edge radius, a negative effective rake angle can be achieved even if a 0° rake angle tool is used. About the effect of cutting edge radius on surface roughness, Yue et al. [39] found that sharp diamond tools produce a smooth surface with small roughness in diamond turning of MgAl_2_O_4_ spinel ceramic.

In terms of the cutting edge quality, many researchers focused on the regular tool wear during processing [40,41] or the corresponding suppression method [42]. Few studies on the micro defects of a cutting edge have been found. By using a diamond edge artifact as a reference, Chen et al. [43] measured the cutting edge chipping and tool wear of the diamond tool used in a force sensor integrated fast tool servo (FS-FTS) assembled on an ultra-precision turning machine. Unfortunately, the accuracy of the proposed on-machine system is only on the order of sub-micron.

As a typical brittle material, potassium dihydrogen phosphate (KDP) crystal is recognized as a difficult-to-process material. Fuchs et al. [44] firstly employed single-point diamond turning (SPDT) to process the KDP crystal and claimed that negative rake tool will generate smaller subsurface damage on the finished surface. Montesanti et al. [45] reported the procedure of diamond turning KDP crystal, in which tool geometries (including tool rake angle and tool nose radius) and tool-edge sharpness were discussed in detail. They concluded that the edge quality is one of the least understood aspects in KDP processing. Their experience showed that even if two diamond tools are obviously the same under the optical microscope, the finally processed surface quality may be quite different, because the tool-edge sharpness and nanoscale micro defects cannot be evaluated. Moreover, Zhang et al. [46] found that the hydrostatic pressure in the cutting area is very important for achieving the ductile-mode machining of KDP crystal in diamond turning. As for the physical surface effects in the microcutting of brittle materials, Lee et al. [47] discussed a novel method named solid coating to enhance the machinability of calcium fluoride (CaF_2_) single crystals in terms of the positive improvements in ductile–brittle transition (DBT).

In terms of the literature reviewed above, it can be found that little progress has been made with regard to the tool-edge micro defects and its corresponding elimination method. However, it is a non-negligible factor that influences the machined surface quality, especially for brittle materials. Therefore, in the Methods part of this work, the tool-edge micro defects at the nanometer level are detected by AFM and classified into three main types according to their different cross-section profiles in Section 2.1. Moreover, the smoothed particle hydrodynamics (SPH) cutting models of tool-edge micro defects are constructed in Section 2.2. The tool-edge passivation method and fly-cutting experiments setup are described in Section 2.3 and Section 2.4, respectively. In the Results and Discussion sections, by using the SPH simulation method, the effect of tool-edge micro defects on the machined surface quality of brittle material, i.e., KDP crystal, is investigated in Section 3.1. Moreover, for keeping the cutting edge as sharp as possible while removing the tool-edge micro defects completely by passivation, the effect of tool shank depression angles on the geometries of the passivated cutting edge is discussed in Section 3.2, and a micro chamfered edge is finally formed through the re-sharpening process of rake face to remove the rake wear after passivation. At last, in Section 3.3, fly-cutting experiments of KDP crystal are carried out to validate the improvement of tool cutting performance after the tool-edge passivation.

## 2. Methods

### 2.1. Detection and Classification of Tool-Edge Micro Defects

**Figure 1:**
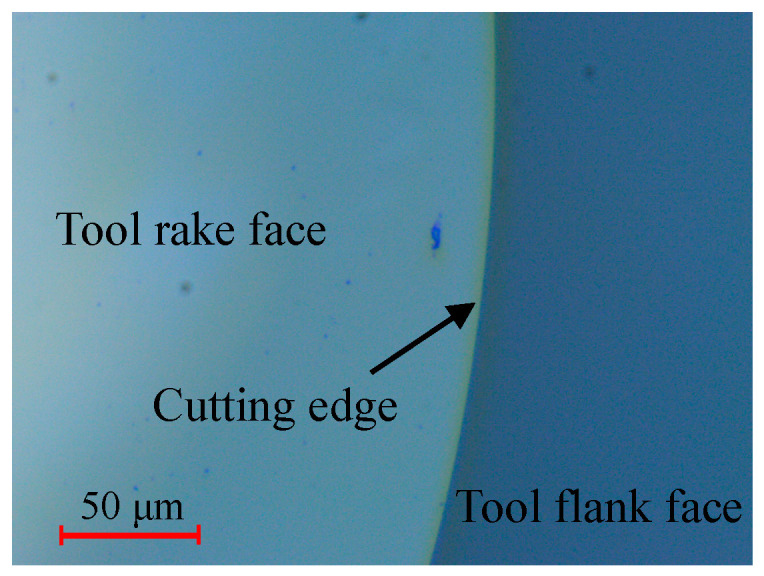
Check of the defects-controlled cutting edge by an optical microscope at 1250 magnification.

In general, the defects-controlled mechanical polishing process of a diamond tool consists of two steps, i.e., tool flank face polishing and tool rake face polishing in sequence. As presented in Figure 1, after a fine polishing of the tool rake face, the tool is observed by an optical microscope at 1250 magnification to ensure that there are no visible defects on the cutting edge. However, due to the natural disadvantage of mechanical polishing especially for brittle materials, small-size defects at the nanometer level that cannot be detected by an optical microscope are bound to occur on the cutting edge, which are named tool-edge micro defects in this work.

**Figure 2:**
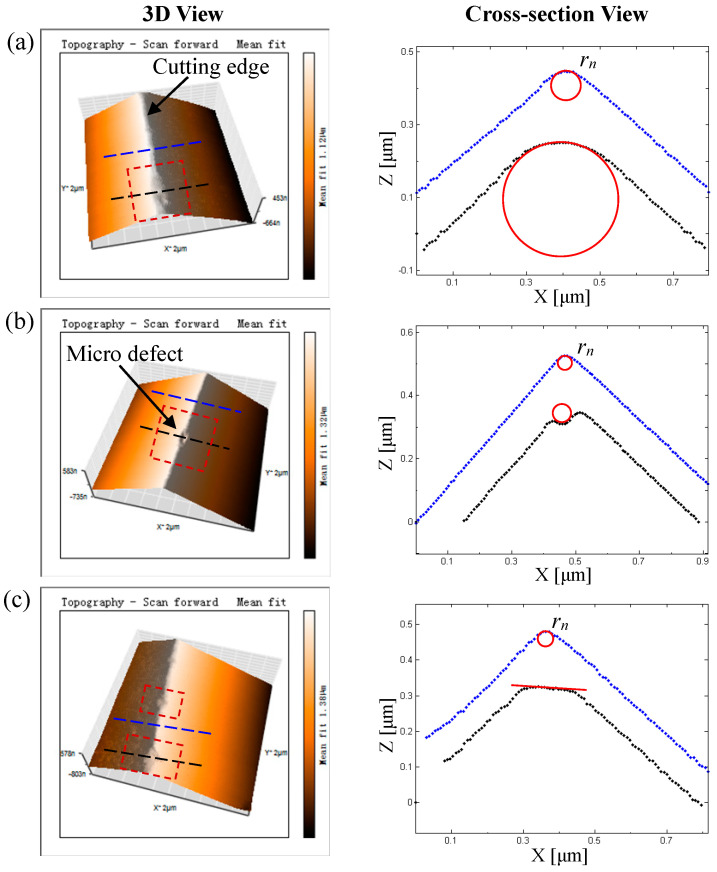
Three types of the tool-edge micro defects measured by atomic force microscopy (AFM) in 3D view and its corresponding cross-section: (**a**) blunt-edge micro defect; (**b**) crescent-edge micro defect; (**c**) flat-edge micro defect.

In order to detect the tool-edge micro defects, the AFM (NaniteAFM, supplied by Nanosurf AG, Liestal, Switzerland) was employed to directly scan a small-size region of the cutting edge in the contact mode. To ensure the accuracy of measurement results, sharp silicon probes with a radius less than 10 nm are used (Stat0.2LAuD-10, supplied by Nanosurf AG). Relying on the defects-controlled manufacturing process, a large number of polished diamond tools were supplied for the AFM detection work of tool-edge micro defects. As a result, three types of tool-edge micro defects are summarized in Figure 2 according to their cross-section profiles. The left images show the measured 3D results. It can be seen that the cutting edge is sharp, but on which there are micro defects distributed, as marked by the red dotted box. The right images show the cross-section views of the cutting edge along the blue and black dotted lines as marked in the 3D views. Obviously, compared with the blue cross-sections of the near-perfect cutting edge, the black cross-sections with micro defects are no longer sharp. According to their shapes, the tool-edge micro defects are classified into three types in this work, including blunt-edge micro defects, crescent-edge micro defects, and flat-edge micro defects. It is worth mentioning that for a specific micro defect, its cross-section will not be exactly the same size, but its shape is similar and definitely belongs to one of these three types. This implies that the classification of tool-edge micro defects in this section is applicable and representative for the diamond tools manufactured by the defects-controlled mechanical polishing process, which inspires us to reveal the underlying mechanism of tool-edge micro defects in machining by a simulation technology, as elaborated in the following text.

### 2.2. SPH Cutting Models of Tool-Edge Micro Defects

**Figure 3:**
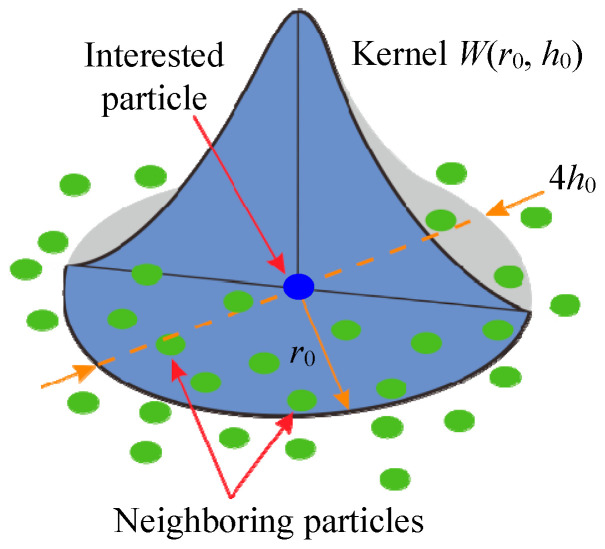
Schematic diagram for the kemel approximation in the smoothed particle hydrodynamics (SPH) method [49].

The SPH method is a mesh-free finite element method, which was first proposed by Gingold and Monaghan in 1977 [48]. In the early stage, the SPH method was only applied in astrophysics. With the development of computer science, its application area had expanded to the continuous solid mechanics. Compared with the traditional method using grids, the SPH method has advantages in dealing with the problems undergoing large deformations such as cutting, because it does not have to solve the problems such as mesh tangling encountered in the extremely large deformation. Figure 3 illustrates the kernel approximation, which is used as a calculation method in SPH. *r*_0_ represents the influence radius of the approximation field for the interested particle. The mathematical formula of particle approximation can be expressed as [49]

(1) $$f\left(x\right)=\sum_{j=1}^{n}\frac{m_{j}}{\rho_{j}}f_{j}W\left(\left|x-x_{j}\right|,h_{0}\right)$$

where *ρ_j_* and *m_j_* represent the density and mass of the neighboring particles, respectively. *x_j_* represents the location of particle *j* with its field variable of *f_j_*. *h*_0_ is the smoothing length, which restricts the searching range of the kernel function *W*.

**Figure 4:**
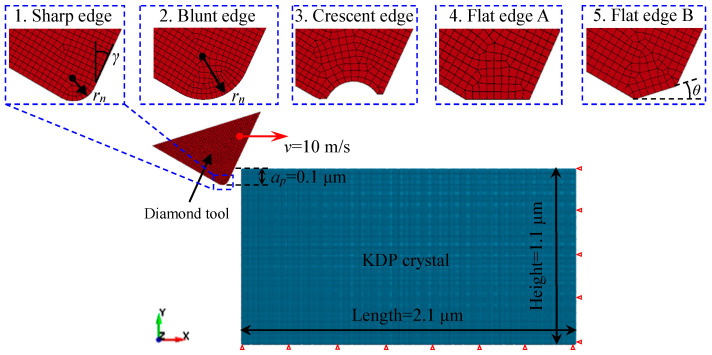
SPH cutting models with different tool-edge micro defects (including sharp edge, blunt edge, crescent edge, flat edge A, and flat edge B).

As shown in Figure 4, the 2D SPH cutting models with different tool-edge micro defects were constructed in this work. The blue part represents the KDP crystal modeled by 36,294 SPH particles, and the dimensions of which are 2.1 μm in length and 1.1 μm in height. The bottom and right particles of the KDP workpiece are constrained in all directions. The red part represents the diamond tool modeled by shell grids. The contact type between the workpiece and diamond tool is “Node-To-Solid” available in LS-DYNA, in which the master part is the diamond tool and the slave nodes are SPH particles. The friction of the tool–workpiece contact interface is described by the Coulomb model with a friction coefficient of 0.12. In addition, the depth of cut *a_p_* is set to 0.1 μm. The tool cutting velocity *v* is set to 10 m/s along the positive direction of the *x* axis.

As the close-up images presented in Figure 4, besides the sharp edge, another four types of edges with different micro defects were modeled according to the classification of tool-edge micro defects above. Compared with the blunt edge and crescent edge, the situation of the flat edge is more complicated. Two types were further classified on the basis of their states in machining. One is flat edge A, whose edge direction is parallel to the cutting velocity. The other is flat edge B, whose edge direction has an included angle *θ* with the cutting velocity. Therefore, together with the sharp edge, five simulation models including four types of defective edges were established. Except for the variation of tool edges, all the other settings in the simulation remain unchanged.

**Figure 5:**
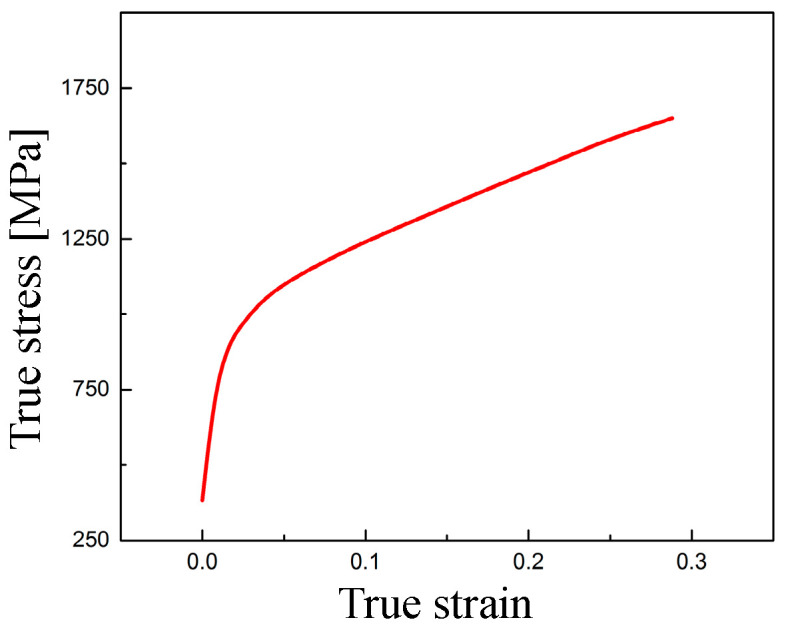
The curve of true stress vs. true strain for KDP crystal in SPH simulations [46].

In addition, since the hardness of diamond is much greater than that of KDP crystal, the diamond tool was regarded as a rigid body in simulation. Considering the soft and brittle characteristics of KDP crystal related to the hydrostatic pressure, a modified material model was constructed in this work, which integrates the elastoplastic model defined by the stress vs. strain curve and the minimum pressure failure criterion. Table 1 tabulates the parameters of material model for KDP crystal, and the curve of true stress vs. true strain is illustrated in Figure 5 [46]. In terms of the minimum pressure failure criterion, when the pressure of SPH particles is below −500 MPa, these particles will be deactivated; that is, the stress states of the deactivated SPH particles will be set to 0. Moreover, the contact defined for SPH also becomes inactive for these deactivated particles.

### 2.3. Tool-Edge Passivation Method

**Figure 6:**
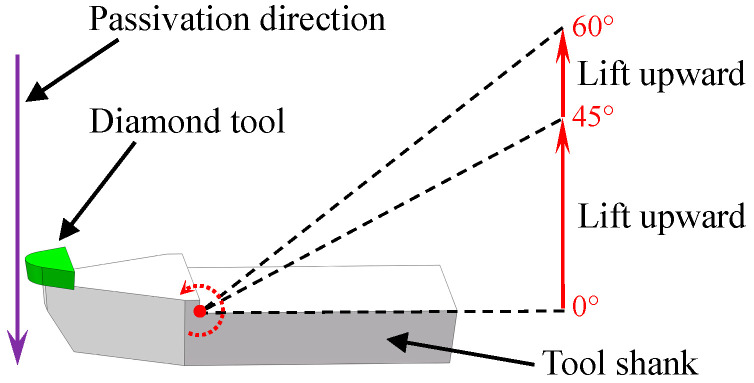
Schematic diagram for the tool-edge passivation method at different depression angles, i.e., 0°, 45°, and 60°.

In the passivation process, a soft pad was used to contact with the cutting edge. In the traditional method, the passivation direction is perpendicular to the tool rake face; in other words, the depression angle of the tool shank is 0°. In this work, in order to obtain a passivated cutting edge for the ductile-mode machining of KDP crystal with satisfactory surface roughness, the tool shank was lifted upward at different angles, i.e., 45° and 60° as illustrated in Figure 6, to study the effect of tool shank depression angles on the geometries of the cutting edge after passivation.

### 2.4. Fly-Cutting Experiments Setup

**Figure 7:**
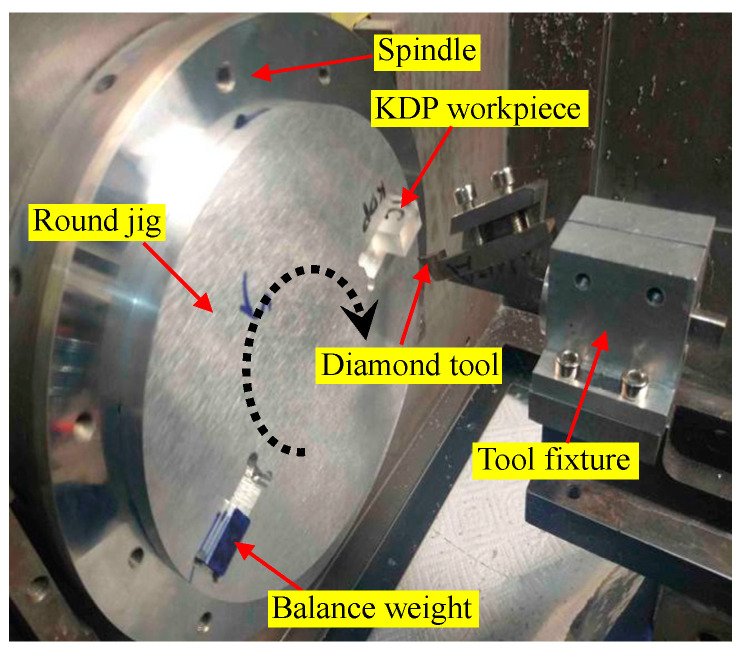
Experimental setup for the fly-cutting of KDP crystal (the balance weight and KDP crystal are symmetrically glued onto the round jig to relieve the effect of material anisotropy on the machined surface quality).

In order to investigate the effect of tool-edge passivation on the machined surface quality, fly-cutting experiments of KDP crystal were carried out on a commercial available machine tool (Nanotech 350FG, supplied by Moore Tools Co., Bridgeport, CT, USA). As shown in Figure 7, in order to relieve the effect of material anisotropy on the machined surface quality, the balance weight and KDP crystal were symmetrically glued onto the round jig. Since the radius of the round jig (i.e., 75 mm) is much greater than the workpiece surface size (i.e., 15 mm in width and 10 mm in length), the cutting paths can be approximately seen as straight lines with no change of the crystal orientation.

Table 2 tabulates the processing conditions used in the fly-cutting experiments. In the test, the cutting parameters remained unchanged, and no cutting fluid was used to avoid the deliquescence. Moreover, to prevent the interferences from other influencing factors, only one tool was used to ensure the same rake angle, relief angle, tool nose radius, and edge waviness. The rake angle of −25° was obtained by using a special tool fixture. Except for the uniform cutting parameters, the details of different tool-edge qualities, i.e., T0, T1, T2, and T3, are elaborated in the following Section 3.2.

## 3. Results and Discussions

### 3.1. SPH Simulation Analysis

**Figure 8:**
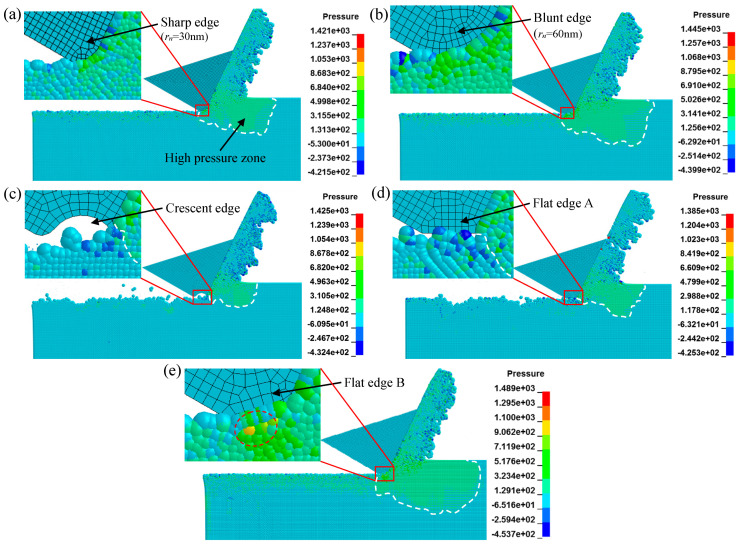
Hydrostatic pressure distribution in the cutting simulations of different types of cutting edge: (**a**) sharp edge with *r_n_* = 30 nm, i.e., the normal group simulation; (**b**) blunt edge with *r_n_* = 60 nm; (**c**) crescent edge; (**d**) flat edge A; and (**e**) flat edge B.

The cutting simulations with different tool-edge micro defects were performed in sequence by the general-purpose program LS-DYNA. In order to investigate the effect of blunt-edge micro defects on the machined surface quality, as shown in Figure 8a,b, two groups of cutting simulations were performed, i.e., the normal group simulation with a sharp edge *r_n_* = 30 nm and the blunt-edge simulation with *r_n_* = 60 nm. It can be seen that the hydrostatic pressure distribution is basically the same at different cutting edge radii. As highlighted by the white dotted lines, a high-pressure zone filled with green particles is formed beneath and in front of the cutting edge, which is beneficial to suppress the cracks formation in cutting. There is a small difference in that the area and density of the high-pressure zone generated in the blunt-edge simulation are slightly larger than the normal group with a sharp edge.

**Figure 9:**
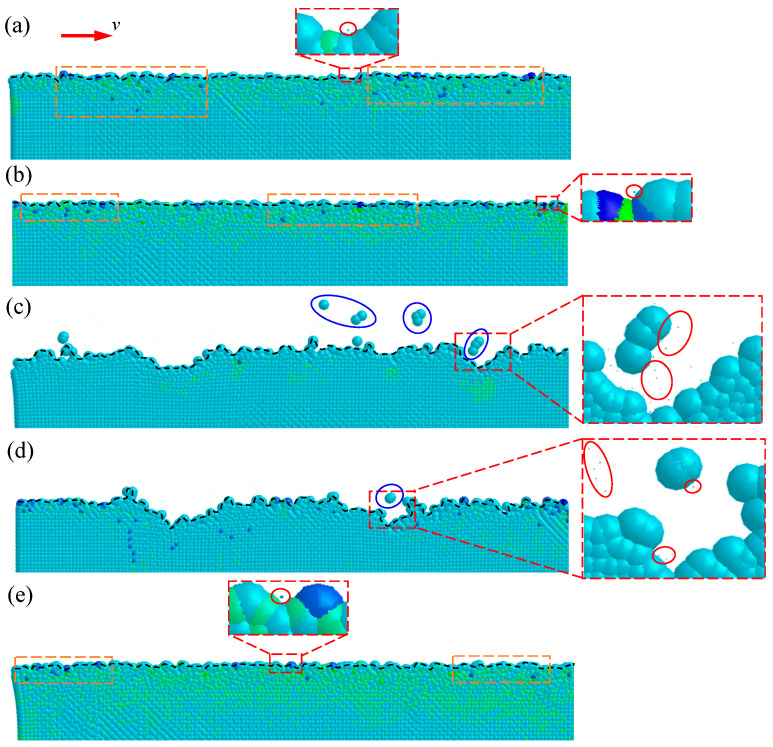
Machined surface in the cutting simulations of different types of cutting edge: (**a**) sharp edge with *r_n_* = 30 nm; (**b**) blunt edge with *r_n_* = 60 nm; (**c**) crescent edge; (**d**) flat edge A; (**e**) flat edge B (with the same stress legend of Figure 8).

In order to investigate the effect of a crescent-edge micro defect on the machined surface quality, as shown in Figure 8c, a new cutting model with the defective tool, i.e., crescent edge, was established based on the sharp edge in the normal group. It can be seen that the distribution of hydrostatic pressure is changed as compared with the normal group simulation. The high-pressure zone only appears in front of the cutting edge. As presented in the close-up, the high-pressure area beneath the cutting edge is no longer distributed, which is due to the disappearance of the burnishing effect caused by the crescent-edge micro defect. Instead, some dark blue particles with large hydrostatic tensile stress emerge in the cutting area. As the cutting tool advances, these dark blue particles will be further tensioned and eventually lead to failure. As shown in the close-up of Figure 9c, many deactivated particles marked by red circles are finally generated, which makes the machined surface unsmooth and large-size defects appear.

In order to investigate the effect of the flat-edge micro defect on the machined surface quality, as shown in Figure 8d,e, two new cutting models with different types of defective tools, i.e., flat edge A and flat edge B, were established based on the sharp edge in the normal group. Viewing from the state in cutting, flat edge A and flat edge B are equivalent to the flank wear and the chamfered cutting edge [50], respectively. The distribution of hydrostatic pressure is visibly different. Compared with the normal group simulation, the high-pressure zone in the simulation of flat edge A only appears in front of the cutting edge. As presented in the close-up of Figure 8d, the high-pressure area beneath the cutting edge is no longer distributed, which is also owing to the decline of the burnishing effect under flat edge A. Instead, some dark blue particles with hydrostatic tensile stress emerge in the cutting area as the same as the crescent-edge simulation. With the advance of cutting tool, flat edge A will rub the machined surface excessively, which makes these dark blue particles further tensioned and eventually deactivated. As shown in the close-up of Figure 9d, many deactivated particles marked by red circles are finally generated, which makes the machined surface unsmooth, and large-size defects appear, too. However, as presented in Figure 8e, the hydrostatic pressure distribution in the simulation of flat edge B is very similar to that observed in the normal group simulation with a sharp edge. The high-pressure zone is formed beneath and in front of the cutting edge. There is a small difference in that the area and density of the high-pressure zone generated by flat edge B are slightly larger than that produced in the normal group simulation. Moreover, as shown in the close-up, even the yellow particle with large hydrostatic compressive stress emerges beneath the chamfered edge, which can sufficiently suppress the cracks formation in cutting.

The machined surfaces in simulations are clearly presented in Figure 9, with the same stress legend of Figure 8. The topmost particles as marked by black dotted lines represent the finally machined surface in simulations. As shown in Figure 9a with the sharp edge, Figure 9b with the blunt edge, and Figure 9e with flat edge B, the surface stress states are all in compression because of the distribution of many green particles with hydrostatic compressive stress. As marked by the orange dotted box, there are a few dark blue particles with hydrostatic tensile stress distributed on the machined surface. Compared with the normal group simulation, the area of green particles is slightly increased and the depth of dark blue particles is slightly reduced in the simulations with blunt edge and flat edge B, which is more helpful to suppress the cracks formation in the machining of brittle materials. Moreover, as demonstrated in the close-ups of Figure 9a,b,e, the points highlighted by red circles represent the deactivated particles that exceed the minimum failure pressure, which are rarely distributed on the surface. As a result, the machined surfaces are overall smooth with a few tiny defects because of the sporadic deactivated particles.

In contrast, as presented in Figure 9c with a crescent edge and Figure 9d with flat edge A, the machined surfaces are no longer in a compression state because the green particles with hydrostatic compressive stress are not densely distributed on the topmost surface and subsurface layer, which results in the brittle-mode removal of materials. As shown in the close-ups, a lot of deactivated particles marked by red circles form the large-size defects on the finally machined surface. In addition, as highlighted by blue circles, the debris made of several active particles is inevitably generated with the formation of large-size defects, which will finally fall and adsorb onto the machined surface and destroy the surface quality.

**Figure 10:**
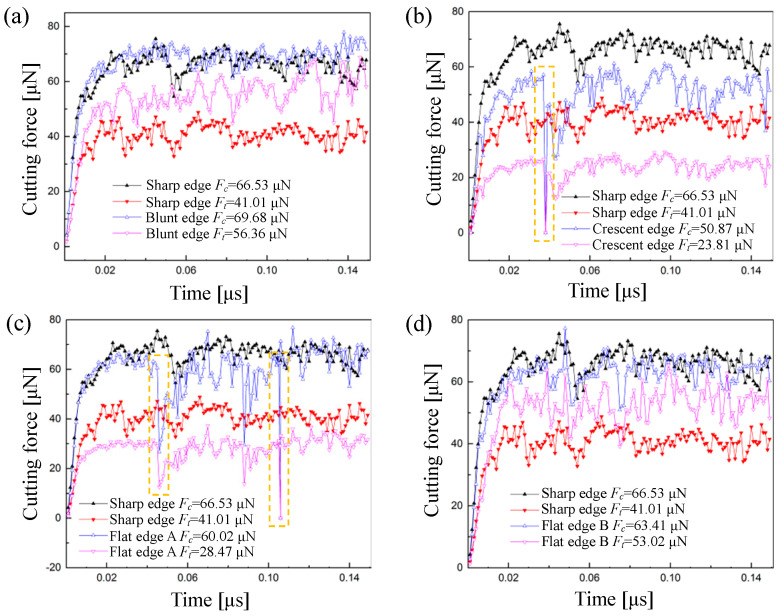
Curves of cutting force vs. time history in simulations: (**a**) comparison of blunt edge and sharp edge; (**b**) comparison of crescent edge and sharp edge; (**c**) comparison of flat edge A and sharp edge; (**d**) comparison of flat edge B and sharp edge (the average results of forces are noted in the legends).

To explore the origin of the variation of hydrostatic pressure distribution in the cutting area and the machined surface layer, the curves of cutting force vs. time history in simulations are plotted in Figure 10, in which the average forces are noted in the legends. As shown in Figure 10a,d, in terms of the principal cutting force *F_c_*, the blunt edge and flat edge B are roughly the same as the normal group simulation using the sharp edge. However, the thrust cutting force *F_t_* is significantly larger than the normal group. The simulation result of the blunt edge is consistent with the findings reported by Hosseini et al. [35], i.e., the ratio of *F_t_*/*F_c_* increases significantly at a small value of *a_p_*/*r_n_*. Obviously, the significantly increased thrust cutting force *F_t_* is the origin of the increase of hydrostatic pressure when the tool has a blunt edge or a flat edge B. The reason is that the blunt tool with a large cutting edge radius makes a large fraction of particles compressed to pass through beneath the edge, which results in the heavy extrusion effect on the materials and the increase of hydrostatic pressure in the cutting area. As for flat edge B, it is mainly caused by the increase of the localized compression effect induced by the micro chamfered edge. Considering the suppression of hydrostatic pressure on the cracks formation [51], the blunt edge and the chamfered edge are more suitable for achieving the defect-free surface of brittle materials.

As shown in Figure 10b,c, the cutting forces of the crescent edge and flat edge A obviously decrease due to the brittle-mode removal of materials. Especially for the thrust cutting force *F_t_*, such a great reduction comes from the disappearance of the squeezing effect on workpiece caused by the crescent-edge micro defect or the flat-edge A micro defect, which is not conducive to the formation of hydrostatic pressure. Moreover, it should be noted that the cutting force in response to the crescent edge and flat edge A will suddenly drop, nearly to zero, in a short time as marked by the yellow dotted boxes in Figure 10b,c. This phenomenon is caused by a temporary non-contact state between the cutting edge and the workpiece when a large-size defect occurs, as demonstrated in Figure 9c,d.

### 3.2. Tool-Edge Passivation Results

The above simulation results reveal that the crescent-edge and flat-edge A micro defects on the cutting edge will destroy the hydrostatic compressive stress state in cutting of brittle materials and produce large-size defects on the finally machined surface. However, no defects are found on the machined surface using an increased cutting edge radius (i.e., the blunt edge in simulation), which indicates a solution for the elimination of tool-edge micro defects, i.e., the tool-edge passivation method. On one hand, the passivation process will eliminate the tool-edge micro defects such as the types of crescent edge and flat edge A, and it will also improve the consistency of cutting edge quality. On the other hand, the passivation method is a flexible removal process by using a soft pad, which will inevitably increase the cutting edge radius after passivation. However, this variation does not produce new defects on the machined surface, which is acceptable for the processing of brittle materials. Moreover, the simulation of flat edge B provides an idea for manufacturing the diamond tool with a novel edge shape, i.e., the micro chamfered edge, for suppressing the cracks formation in the cutting area efficiently.

**Figure 11:**
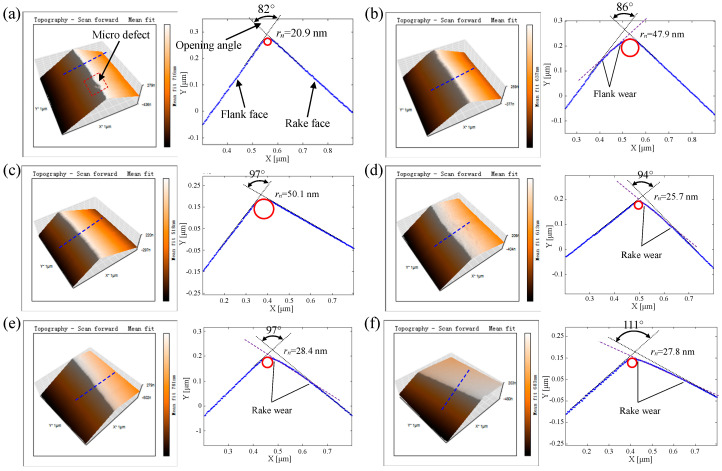
Comparison of the cutting edge quality before and after the tool-edge passivation: (**a**) before passivation; (**b**) after the 2-min traditional passivation method; (**c**) after the 2-min passivation at the depression angle of 45°; (**d**) after the 2-min passivation at the depression angle of 60°; (**e**) after the 4-min passivation at the depression angle of 60°; (**f**) after the 6-min passivation at the depression angle of 60°.

In addition, considering that the large cutting edge radius promotes the plastic side flow and increases the height of tool marks [52,53], the goal of the tool-edge passivation process should be keeping the cutting edge as sharp as possible while removing the tool-edge micro defects completely. Figure 11a shows the measured result of the sharp edge before passivation; obviously, there is a micro defect, as marked by the red box on the cutting edge. The right image is the cross-section view of the cutting edge along the blue dotted line as marked in the left image, in which the flank face is placed uniformly on the left and the rake face is on the right. The opening angle between the rake face and the flank face is 82°, and the cutting edge radius is 20.9 nm, which is fitted by the least square arc method. By using the traditional passivation method at a depression angle of 0°, the tool cutting edge after 2-min passivation is measured by AFM, as presented in Figure 11b. Compared with the sharp edge, the cutting edge after the traditional passivation process is smooth and the micro defect is removed, but the opening angle and cutting edge radius consequentially increase to 86° and 47.9 nm, respectively. Moreover, a flank wear is found nearby the cutting edge. Figure 11c presents the measured result of the cutting edge after a 2-min passivation process at a depression angle of 45°. The opening angle and cutting edge radius also increase to 97° and 50.1 nm, respectively. However, there is no flank wear found nearby the cutting edge. Figure 11d is the measured result of the cutting edge after a 2-min passivation process at a depression angle of 60°. In addition to the increased opening angle, the cutting edge radius is only 25.7 nm with a small increment compared with the depression angles of 45° and 0°. Moreover, a rake wear is formed, because the rake face materials nearby the cutting edge are removed more during the passivation. Subsequently, the cutting edge is passivated at the depression angle of 60° for a longer time, and the measured results of the cutting edge after 4-min and 6-min passivation are presented in Figure 11e,f, respectively. One interesting finding is that the cutting edge radius does not increase with the passivation time, but the consistency of cutting edge quality becomes better, which perfectly satisfies the passivation goal. Another change is that the opening angle increases with the passivation time, which means that more and more materials on the rake face are removed in passivation.

**Figure 12:**
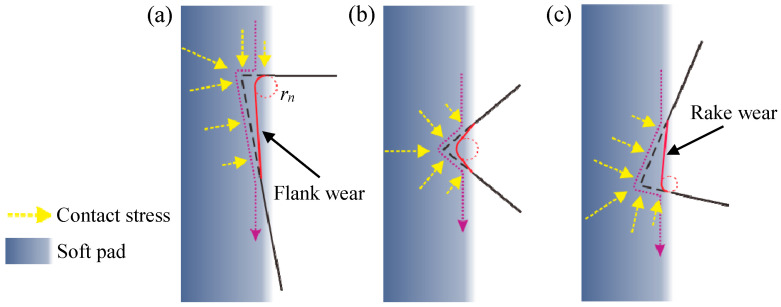
Schematic diagram of the material removal mechanism in tool-edge passivation process at different depression angles: (**a**) 0°; (**b**) 45°; (**c**) 60°.

The passivation results above have indicated that the depression angle has a great influence on the geometries of tool cutting edge after passivation. Considering that the passivation method is a flexible removal process, the material removal mechanism is proposed to reveal the contact stress between the soft pad and the cutting edge. As illustrated in Figure 12, the cyan part with color gradients represent the soft pad. The yellow dotted arrows represent the contact stress at the interface between the soft pad and the cutting edge, the length of which qualitatively represents the magnitude of the contact stress. At the depression angle of 0° as shown in Figure 12a, the black dotted line represents the sharp edge pressing into the soft pad, and the purple dotted arrow represents the materials flow of the soft pad under the contact stress. Affected by the direct impact on the rake face and the varying contact stress determined by the pressing depth, different material removal efficiency results in the shape of cutting edge after passivation, as shown by the red curve. The flank wear is formed because of the excessively friction on the flank face, and the cutting edge radius is increased due to the direct impact. When the depression angle is 45° as presented in Figure 12b, the cutting edge almost symmetrically presses into the soft pad, which makes the removal efficiency symmetrically distributed on the rake and flank faces, and none of them is excessively worn. However, when the depression angle is 60° as shown in Figure 12c, the rake wear is formed due to the excessively friction on the rake face. As the material on the rake face is gradually removed, the passivated rake face is gradually parallel to the soft pad, which makes the cutting edge radius no longer subject to the direct impact. This is the reason why the radius of the cutting edge in passivation with a depression angle of 60° does not increase with time.

**Figure 13:**
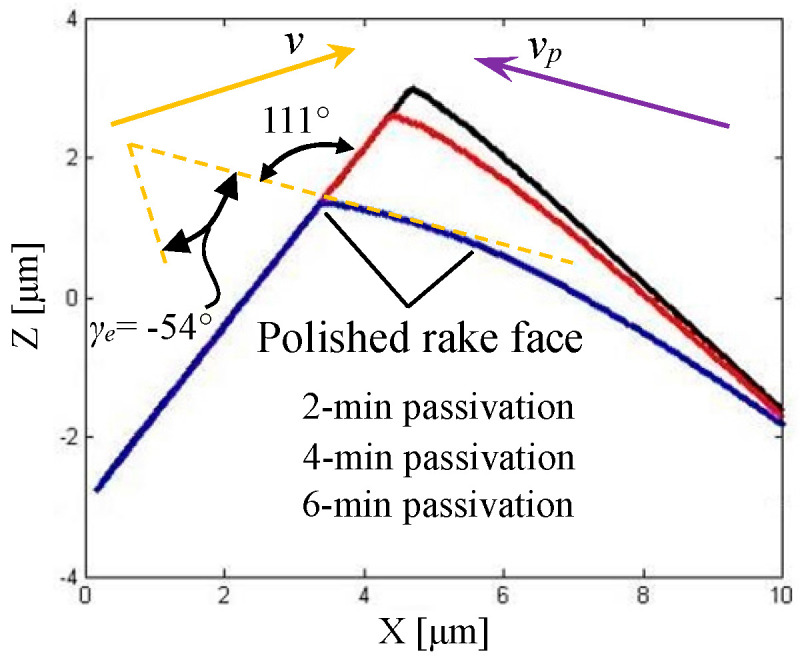
Large-size 2D scan results of the cutting edge after different passivation times (*v_p_* represents the soft pad velocity, *v* represents the tool cutting velocity).

As presented in Figure 13, the large-size 2D scan results of the cutting edge indicate that the rake face material nearby the cutting edge is increasingly removed with the passivation time. When the passivation time is 6 min, the opening angle of the passivated cutting edge is 111°, which makes the passivated rake face near the cutting edge approximately parallel to the soft pad velocity *v_p_*, i.e., the purple arrow in the image. Such an observation well verifies the correctness of the proposed material removal mechanism in passivation with a depression angle of 60°.

**Figure 14:**
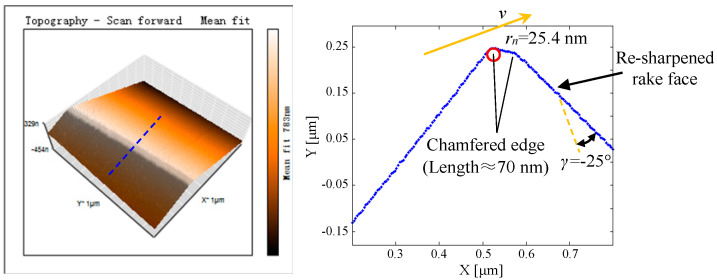
Measured result of the chamfered cutting edge after the passivation at the depression angle of 60° and the re-sharpening of rake face (*v* represents the tool cutting velocity).

From the passivation results at the depression angle of 60°, it can be found that while removing the tool-edge micro defects completely and improving the consistency of cutting edge quality, a sufficient sharpness can be achieved for the cutting edge radius. However, a rake wear is inevitably formed, which results in the increment of opening angle. Moreover, due to the flexible removal characteristics of passivation, the worn rake face in the section view of Figure 13 is not straight but has a small curvature. To fabricate a chamfered edge such as flat edge B in the simulation, after 6-min passivation at a depression angle of 60°, the rake face is re-sharpened to remove the rake wear. As presented in Figure 14, the orange arrow represents the tool cutting velocity *v*, and a micro chamfered edge about 70 nm in length is finally formed. Meanwhile, because of the previous passivation procedure at the depression angle of 60°, the tool-edge quality is satisfied without micro defects, the sharpness of which is only 25.4 nm.

For achieving the ductile-mode machining of brittle materials, in this work fly-cutting experiments of KDP crystals were performed by using the diamond tool with different cutting edge qualities. For example, the tool-edge quality T0 represents the freshly sharpened tool with tool-edge micro defects, as presented in Figure 11a. The tool-edge qualities T1 and T2 represent the passivated cutting edges at the depression angles of 0° and 60°, as presented in Figure 11b,f, respectively. Moreover, the tool-edge quality T3 represents the chamfered cutting edge after passivation at a depression angle of 60° and re-sharpening of the rake face, as presented in Figure 14.

### 3.3. Machined Surface Quality in Fly-Cutting Experiment

**Figure 15:**
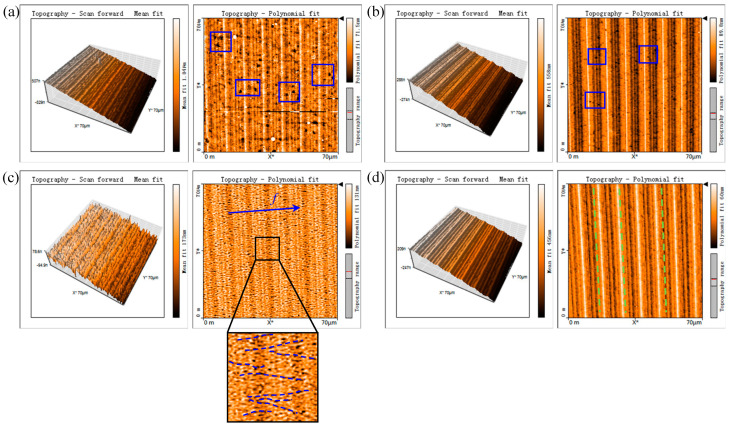
Machined surface in fly-cutting experiments of KDP crystal by different tool-edge qualities: (**a**) T0, freshly sharpened tool with tool-edge micro defects; (**b**) T1, passivated cutting edge in the traditional method at a depression angle of 0°; (**c**) T2, passivated cutting edge at a depression angle of 60°; (**d**) T3, chamfered cutting edge after passivation at a depression angle of 60° and re-sharpening of rake face.

After fly-cutting experiments, the machined surface was measured by AFM with a scanning area of 70 × 70 μm^2^. As presented in Figure 15, the left images are 3D views, and the right images are 2D views. When the new tool with tool-edge micro defects is used, as highlighted by the blue box in Figure 15a, many large-size defects appear on the machined surface. However, when the tool is passivated by the traditional method, significant improvement in the condition of surface defects can be seen in Figure 15b. Only some small-size defects are distributed on the machined surface due to the suppression of cracks by the increased cutting edge radius. Figure 15c presents the machined surface by the cutting edge passivated at a depression angle of 60°. Although there are no large-size defects such as those in Figure 15a, a lot of microcracks along the tool feeding direction spread over the machined surface. In addition, the tool marks left on the machined surface are not visible as compared with others. The reason is that the passivated cutting edge is hidden back because of the appearance of rake wear. As shown in Figure 11f, although the cutting edge is perfect and sharp after passivation with a depression angle of 60°, the rake wear makes the cutting edge have an effective rake angle *γ_e_* in machining, which is extremely negative, i.e., −54°, as shown in Figure 13. Moreover, the worn rake face with a small curvature also acts as a very blunt edge, which excessively burnishes and rubs the machined surface together with the extremely negative rake angle. Thus, instead of generating the large-size defects shown in Figure 15a, microcracks easily form along the tool feeding direction. Figure 15d presents the machined surface by the chamfered cutting edge. Obviously, there are no defects due to the local hydrostatic compressive stress induced by the micro chamfered edge. Moreover, microcracks along the tool feeding direction also disappear because the rake wear formed in passivation has been removed in the re-sharpening process of rake face. As illustrated in Figure 14, the re-sharpened rake face forms a regular negative rake angle of −25° with the normal direction of cutting velocity *v*.

**Figure 16:**
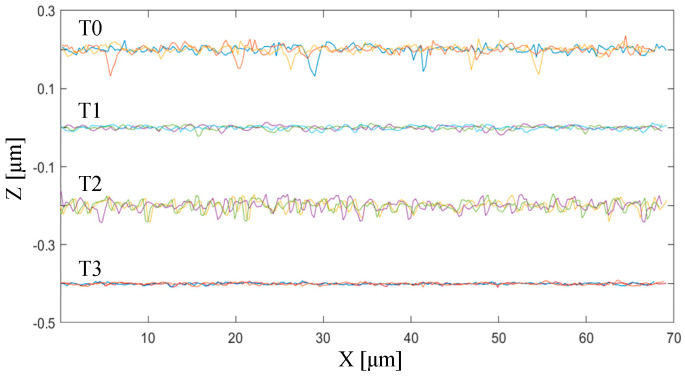
Machined surface topographies along the cutting paths extracted from Figure 15 by different tool-edge qualities (T0, T1, T2, and T3).

As marked by the green dotted lines in Figure 15d, the machined surface topographies along three cutting paths are extracted and plotted in Figure 16. Compared with the new tool T0, the surface topography achieved by the passivated edge T1 is much smoother with small-size defects. On the contrary, the surface topography finished with the cutting edge passivated at a depression angle of 60° is still unsmooth with a lot of defects induced by the widespread microcracks along the tool feeding direction. As the same as simulation results with a micro chamfered edge (i.e., the flat edge B), the surface topography machined by the re-sharpened edge T3 is very smooth with few micro defects.

**Figure 17:**
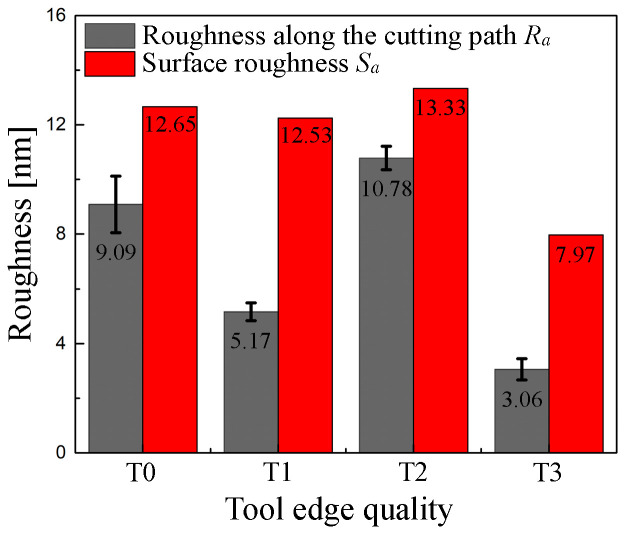
Measured roughness of the machined surface in response to different tool-edge qualities.

The roughness of the machined surface in response to different tool-edge qualities is presented in Figure 17. The line roughness *R_a_* and area roughness *S_a_* are calculated by the following formulas

(2) $$\left\{ \begin{array}{l} R_{a}=\frac{1}{N}\sum_{l=0}^{N-1}\left|z(x_{l})-z_{m}\right|\\ S_{a}=\frac{1}{MN}\sum_{k=0}^{M-1}\sum_{l=0}^{N-1}\left|z(x_{k},y_{l})\right| \end{array}\right.$$

where *z_m_* represents the mean value, which can be expressed as

(3) $$z_{m}=\frac{1}{N}\sum_{l=0}^{N-1}z(x_{l})$$

Compared with the tool-edge quality T0, the roughness *R_a_* acquired along the cutting path by T1 is significantly reduced. However, the surface roughness *S_a_* remains basically unchanged, which can be attributed to the increment of plastic side flow promoted by the enlarged cutting edge radius using the traditional passivation method. Regardless of roughness *R_a_* along the cutting path or surface roughness *S_a_*, the surface processed by T2 is the largest. This means that although the cutting edge passivated at a depression angle of 60° remains sharp, the worn rake face extremely rubs the machined surface and finally destroys the surface with microcracks. When the rake wear is removed by the re-sharpening process of rake face, the chamfered edge T3 finishes the best surface. On one hand, the roughness *R_a_* along the cutting path is visibly reduced to 3.06 nm because the defects are well-suppressed by the localized hydrostatic compressive stress beneath the micro chamfered edge. On the other hand, the tool-edge quality is perfect without micro defects and retains sufficient sharpness because of the new passivation method at a depression angle of 60°, which ensures that the minimum chip thickness remains small and the plastic side flow does not increase greatly. Finally, a satisfactory surface roughness *S_a_* = 7.97 nm is achieved in this case.

## 4. Conclusions

In this work, based on the classification of tool-edge micro defects, the SPH method is employed to reveal the material removal mechanism of KDP crystal when the diamond tool has different types of tool-edge micro defects. Moreover, the passivation technology is also employed to eliminate the tool-edge micro defects. To keep the cutting edge as sharp as possible while removing the tool-edge micro defects completely after passivation, the effect of depression angle on the geometries of passivated cutting edge is discussed in detail. Finally, the effect of tool-edge passivation on the machined surface quality is validated by fly-cutting experiments of KDP crystal. In light of the findings from the simulation and experiment, some important conclusions can be drawn as follows.

(1) Through AFM detection of the cutting edge of the mechanically polished diamond tool, three types of tool-edge micro defects are classified according to their cross-sections, including the blunt-edge, crescent-edge, and flat-edge micro defects. In addition, the flat-edge micro defects can be further subdivided into flat edge A (similar to flank wear) and flat edge B (similar to chamfered edge) in terms of their states in machining.
(2) The SPH simulation results indicate that the tool-edge micro defects have a great influence on the machined surface quality of KDP crystal. Due to the disappearance of hydrostatic pressure beneath the cutting edge, the machined surfaces of crescent edge and flat edge A are unsmooth with large-size defects. In contrast, the machined surfaces of blunt edge and flat edge B are smooth with a favorable increment of hydrostatic pressure, which indicates that a passivation method to eliminate tool-edge micro defects is necessary.
(3) As revealed by the passivation results, the depression angle of the tool shank has a great influence on the geometries of the passivated cutting edge. Compared with the traditional method with a depression angle of 0°, the passivation results at a depression angle of 60° indicate that the sharpness of the cutting edge is small enough while removing the tool-edge micro defects completely and improving the consistency of the cutting edge quality. Moreover, through the re-sharpening process of the rake face to remove the rake wear, a micro chamfered edge is finally formed, which contributes to suppressing the formation of cracks in machining brittle materials.
(4) Fly-cutting experiments of KDP crystal reveals that the tool-edge quality has a significant effect on the machined surface quality. Compared with the mechanically polished tool with tool-edge micro defects, the cutting edge passivated with the traditional method has a certain suppression effect on the machined surface defects, but the surface roughness *S_a_* varies slightly due to the increment of cutting edge radius. The rake wear formed by the new passivation method at a depression angle of 60° will destroy the machined surface with microcracks. However, the re-sharpening process of the rake face can produce a chamfered edge, which yields the best surface quality. In this case, the defects are well-suppressed by the local hydrostatic compressive stress beneath the micro chamfered edge. Moreover, the sufficient sharpness of the cutting edge is also beneficial to achieving a satisfactory surface roughness.

## Figures and Tables

**Table 1:** Material model parameters for the potassium dihydrogen phosphate (KDP) crystal used in SPH simulations.

Density *ρ* [g/cm^3^]	Elastic Modulus *E* [GPa]	Poisson’s Ratio *v*	True Yield Stress *σ_s-true_* [MPa]	Curve of True Stress vs. True Strain	Minimum Pressure Failure Criterion *σ_h-min_* [MPa]
2.338	53.28	0.26	383.1 [46]	Figure 6	−500

**Table 2:** Processing conditions used in fly-cutting experiments of KDP crystal.

Processing Conditions	Configuration
Depth of cut *a_p_*	3 μm
Spindle speed *n*	1200 rpm
Feed rate *f*	10 μm/r
Cutting fluid	None
Rake angle *γ*	−25°
Tool edge waviness *w*	0.08 μm
Tool nose radius *r_ε_*	0.989 mm
Tool edge quality	T0
	T1
	T2
	T3
